# Laparoscopic Management of Large Ovarian Cysts at a Rural Hospital

**DOI:** 10.4103/0974-1216.71616

**Published:** 2009

**Authors:** Vishwanath V Shindholimath, S G Jyoti, K V Patil, A S Ammanagi

**Affiliations:** Vishwajyoti Nursing Home and Laparoscopic Surgical Centre, APMC Circle, Gokak 591 307, Karnataka

**Keywords:** Laparoscopy, ovarian cyst, rural set-up

## Abstract

**Objective:**

To assess the feasibility and outcome of laparoscopic surgery for the management of large ovarian cysts at a rural hospital.

**Materials and Methods:**

Fifteen patients from March 2004 to February 2007, with large ovarian cysts, with diameter >10 cm, were managed laparoscopically. The masses were cystic and were not associated with ascites or enlarged lymph nodes on ultrasound. Serum CA-125 levels were within the normal range (35 U/ml). Preoperative evaluation included history, clinical examination, sonographic images and serum markers. The management of these ovarian cysts included aspiration, cystectomy or salphingo-oophorectomy, depending on the patient’s age, obstetric history and desire of future fertility. In large, solid, fixed or irregular adnexal masses, suspicious of malignancy, laparotomy was done.

**Results:**

Five patients presented with pain in the abdomen and 10 patients with abdominal distension and discomfort. The average maximum diameter of the ovarian cysts was 16.75 cm (range 10–24 cm). The mean duration of the operation was 80 min. The postoperative hospital stay was from 4 to 6 days. No intraoperative complications occurred and the hospital course of all patients was uncomplicated. In one case, laparoscopy was converted to laparotomy. One patient had minor wound infection at umbilical port site. The patients did not report any complaints during follow up and the clinical examination findings were normal in all, up to 9 months after discharge.

**Conclusion:**

With proper patient selection, the size of an ovarian cyst is not necessarily a contraindication for laparoscopic surgery.

## INTRODUCTION

Laparoscopy has become an accepted method of management of ovarian cysts. Large ovarian cysts, however, continue to be treated by laparotomy. The extremely large ovarian cyst presents a major challenge for the endoscopic surgeon. Increased probability of malignancy, technical problems related to the removal of such cysts and perioperative problems related to cardiorespiratory functional changes may complicate surgery for such cysts.[1] Recent advances in endoscopic surgical techniques have offered new possibilities for laparoscopic treatment of large ovarian cysts.[2] We report our experience with 15 cases of large ovarian cysts, which were managed laparoscopically, in a rural set-up.

### Aims and objectives

The aim of the study was to assess the feasibility and outcome of laparoscopic surgery for the management of large ovarian cysts at a rural hospital and to encourage laparoscopy for the treatment of such ovarian cysts.

## MATERIALS AND METHODS

In a retrospective study, we evaluated 15 patients with large ovarian cysts, with low probability for malignancy, treated laparoscopically in our hospital from March 2004 to February 2007.

Inclusion criteria included large ovarian cyst with size more than 10 cm, absence of ascites, absence of complex mass, no evidence of intraperitoneal spread, no enlarged pelvic lymph nodes and normal serum tumor marker levels (normal serum CA-125 < 35 U/ml). In each case, informed consent was obtained, including a statement that laparotomy might be required if cancer was suspected intraoperatively or if the mass could not be properly managed by laparoscopy alone. All the operations were performed under general anesthesia.

Preoperative evaluation included history, clinical examination, sonographic images and serum markers. Large, solid, fixed or irregular adnexal masses suspicious of malignancy were not included in this study and were treated by laparotomy. CA-125 estimation was done for all women and 35 U/ml was taken as upper limit.

For peritoneal access, open laparoscopy was chosen in all the cases. An incision of about 1.5 cm at the umbilical or supraumbilical area was made and dissection of abdominal layers was performed under vision until the peritoneal cavity was entered. For extremely large cysts, 5 mm trocar and sleeve were introduced directly into the cyst; trocar was removed, and the contents aspirated with suction cannula.

After the inspection of the pelvis, ovaries, upper abdomen, omentum, liver and diaphragmatic surfaces for any growths or other signs of malignancy, the cyst contents were aspirated under vision with 5 mm suction cannula; once the capsule was opened, the laparoscope was introduced inside the cyst and interior of the capsule was examined for any suspicious areas. Two 5 mm working ports were created on both sides at the level of umbilicus, in the mid clavicular line. The management of the ovarian cysts included aspiration of the fluid content and cystectomy or salphingo-oophorectomy, depending on the patient’s age, obstetric history and desire of future fertility.

In ovarian cystectomy, capsule was stripped from the ovarian stroma using two graspers for traction and counter traction. Bipolar forceps was used to coagulate the bleeding vessels at the base of capsule. In postmenopausal women and in those patients in whom ovary and tube could not be conserved, salphingo-oophorectomy was performed. For removal of ovarian cyst, one of the accessory ports was converted to 10 mm, tissue held with grasper under vision and was pulled out along with cannula.

We followed very strict criteria for preoperative evaluation and on slightest suspicion of the nature of ovarian cysts, laparotomy was preferred. We extracted ovarian cysts without endobags and in spite of minimal spillage of the contents, postoperative outcome was not affected.

After the tissue was removed, the abdominal and pelvic cavities were thoroughly irrigated with copious amount of normal saline. All patients, except for one who had conversion to laparotomy, were discharged on fourth postoperative day.

## RESULTS

The mean age of the patients was 45 ± 20 (range 14–89 years). Three patients were in pediatric age group (<18 years), 11 were premenopausal and 1 was postmenopausal. Five patients presented with pain in the abdomen and 10 patients with abdominal distension and discomfort [Table 1].

**Table 1:** Common symptoms of patients with large ovarian cysts

Symptoms	Patients (*n*)
Abdominal pain	5
Abdominal distension, discomfort	10
Total	15

**Figure 1:**
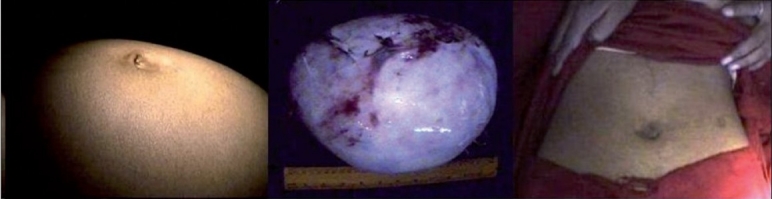
A large ovarian cyst - (a) pre-operative photograph, (b) excised cyst, (c) post-operative photograph

**Figure 2:**
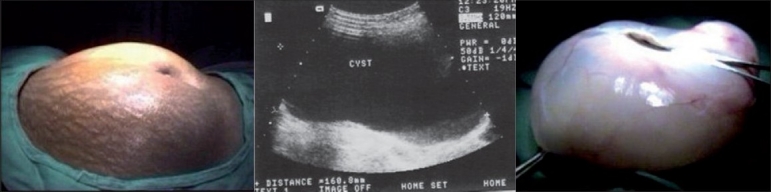
Serous cystadenoma - (a) pre-operative photograph, (b) sonographic picture, (c) excised cyst

The average maximum diameter of the ovarian cysts was 16.75 cm (range 10–24 cm) [Figures 1 and 2]. The patients underwent cystectomy or adnexectomy, depending on each patient’s age and obstetric history [Table 2]. The mean duration of the operation was 80 min (ranged from 60 to 130 min). The postoperative hospital stay was from 4 to 6 days. No intraoperative complications occurred and the hospital course of all patients was uncomplicated. One procedure was converted to laparotomy, because of thick content of the cyst which could not be aspirated with suction cannula.

**Table 2:** Type of laparoscopic surgery

	Patients (*n*)
Laparoscopic cystectomy	5
Laparoscopic oophorectomy	3
Laparoscopic salphingo-oophorectomy	6
Conversion to laparotomy (salphingo-oophorectomy for dermoid cyst)	1
Total	15

All the patients had benign ovarian cysts [Table 3]. The most common histopathologic pattern was that of a serous cystadenoma. All the patients were operated electively; four of them had adnexal torsion. There were no complications and blood loss in all procedures was minimal. The postoperative recovery was uneventful in all women. One patient had minor wound infection at the umbilical port site. The patients did not report any problems during follow up and the clinical examination findings were normal in all up to 9 months after discharge.

**Table 3:** Histopathology of the ovarian cysts

	Patients (*n*)
Serous cystadenoma	8
Mucinous cystadenoma	6
Dermoid cyst	1
Total	15

## DISCUSSION

Operative laparoscopy is regarded today as the gold standard for the surgical treatment of ovarian cysts. Large ovarian cysts, however, continue to be treated by laparotomy. This is mainly due to technical difficulties and the possibility of malignancy. The safety of laparoscopic management of benign adnexal masses has been amply demonstrated. The procedure is associated with reduced operative blood loss, fewer postoperative complications, shorter hospitalization, less pain and earlier recovery compared with laparotomy.[3]

Some surgeons limited laparoscopic surgery to ovarian cyst of size less than 10 cm.[4–7] For apparently benign, extremely large ovarian cysts, only few surgeons advocate laparoscopic management.[6–9] Salem[10] reported 15 cases of large benign ovarian cysts reaching above the level of the umbilicus, which were managed laparoscopically.

One major concern with laparoscopic management of a large ovarian cyst is the possibility of cutting into a malignant neoplasm. This may cause intraperitoneal spillage and trocar site implantation of malignant cells. The possible adverse effect of operative spillage is still controversial. Maiman *et al*,[11] have reported that surgical rupture may unfavorably influence prognosis. However, this has not been confirmed by others using multivariate analysis. In multivariate analysis of stage 1 cancer, factors that influenced the rate of relapse were tumor grade, dense adhesions, and ascites and intraoperative spillage demonstrated no adverse effect on prognosis.[12] Nevertheless, a serious attempt should be made to avoid spillage as much as possible and thorough wash should be given at the end of the procedure.

Port-site metastasis after laparoscopic removal of malignant adnexal tissue is another reported complication, with a reported incidence of 1–16%.[13,14] Extraction of material in endobags is unanimously accepted;[7,15,16] however, we followed strict criteria for preoperative evaluation and on slightest suspicion of nature of ovarian cysts, laparotomy was performed. We extracted ovarian cysts without endobags, and in spite of minimal spillage of the contents, postoperative outcome was not affected. There is no absolute certainty of preventing spillage even with the endobag, since not all endoscopic bags available are of a sufficient quality. The risk of rupture of the various endobags examined differed significantly.[17]

In our small series, we did not have any malignancy. Our experience demonstrates that with proper patient selection, laparoscopy can be applied in the management of a selected group of patients with extremely large ovarian cysts.

## CONCLUSION

With proper patient selection, the size of an ovarian cyst does not necessarily constitute a contraindication for laparoscopic surgery.
